# Survey of injury sources for a trampoline with equipment hazards designed out

**DOI:** 10.1111/j.1440-1754.2012.02426.x

**Published:** 2012-07

**Authors:** David Eager, Carl Scarrott, Jim Nixon, Keith Alexander

**Affiliations:** 1 Faculty of Engineering and Information Technology, University of Technology SydneySydney, New South Wales; 2 Paediatrics and Child Health, University of QueenslandBrisbane, Queensland, Australia; 3 Department of Mathematics and StatisticsChristchurch, New Zealand; 4 Department of Mechanical Engineering, University of CanterburyChristchurch, New Zealand

**Keywords:** child, home, product, trampoline, trampoline safety

## Abstract

**Aim:**

In Australia, trampolines contribute approximately one-quarter of all childhood play-equipment injuries. The purpose of this study was to gather and evaluate injury data from a nontraditional, ‘soft-edged’, consumer trampoline in which the equipment injury sources have been designed out.

**Methods:**

A survey was undertaken in Queensland and New South Wales. The manufacturer of the nontraditional trampoline provided the University of Technology, Sydney, with their Australian customer database. Injury data were gathered in a pilot study by phone interview, then in a full study through an email survey. Results from 3817 respondents were compared with earlier Australian and US data from traditional trampolines gathered from emergency departments.

**Results:**

A significantly lower proportion of the injuries caused by falling off or striking the equipment was found for this new design when compared with traditional trampolines both in Australia and in the USA. The age of children being injured on trampolines in Australia was found to be markedly lower than in North America.

**Conclusions:**

This research indicates that with appropriate design the more severe injuries on traditional trampolines can be significantly reduced.

Numerous studies from a variety of countries have shown that trampoline use can be a potentially dangerous activity for children.^1^–^10^ Typically, these reports show an increasing incidence as sales rise.^4^,^6^,^10^ Alexander *et al*. have reported that almost 50% of injuries from traditional trampolines in the USA result from the trampolines themselves by allowing children to fall off or engage with the frame and springs.^11^,^12^ An Australian paper by Wallis *et al*. from 2004 reports 80% of injuries from the same causes.^13^ In Australia, trampoline-related injuries requiring hospital treatment accounted for 24% of child-related play-equipment falls, an increase of 20% over the decade to 2005.^14^

While it is clear that children are injured on trampolines, it can be argued that regular trampoline use offers health benefits and many parents clearly believe trampolines provide value for their children. These are good reasons to try to improve the safety profile of the trampolines themselves.

In recent years Standards organisations, manufacturers and designers have attempted to reduce trampoline injuries by putting warnings on the equipment and applying layers of protection. This study examines a nontraditional ‘soft-edged’ trampoline that has been designed with the specific goal of removing the hazards altogether by engineering out the equipment-related problem areas.

A soft-edged trampoline is compared with a representative traditional design in Figure 1. Traditional designs have an outer steel frame with springs extending inwards to the jumping surface. Standards require that protective padding should effectively cover the springs and frame. An enclosure net supported by poles is optional. It is common to see traditional trampolines still in use without protective padding and without an enclosure net. The soft-edged trampoline, by contrast, has the resilient jumping surface extending to and including the outer edge so that it needs no protective padding. An enclosure net with flexible poles is integral with the product, and the hard frame is inaccessible to the user.

**Fig. 1:**
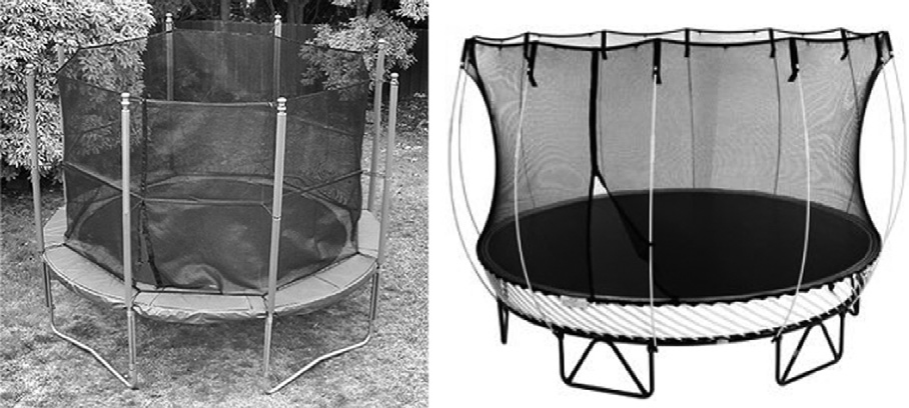
Traditional trampoline (left) with pads covering the frame and springs and padded steel poles holding up the net. A nontraditional, soft-edge trampoline (right) has a resilient mat edge supported by flexible rods, and flexible fibreglass poles tensioning the net.

The primary purpose of this research was to determine whether the design of this soft-edged trampoline has reduced the proportion of trampoline injuries from equipment-based causes.

**Table:** 

What is already known on this topic	What this paper adds
Injuries from consumer trampolines have been well publicised	This study examines a trampoline designed specifically to reduce the injuries, from falling off or impacts with the equipment.
Safety interventions such as pads and enclosures have been required by Standards.	The findings show a significantly lower proportion of injuries from these causes and an overall injury reduction of between 30 and 80% for this design.
There is no evidence in the US data of any reduction in the injuries that should be prevented by the recommended interventions for the period from 2002 to 2008.	Injury rates appear higher in Australia for traditional trampolines, with younger children being injured.

## Methods

The trampoline manufacturer provided a customer database from which trampoline-owning parents were invited to provide information. Stages 1 and 2 of this research were pilot studies seeking an understanding of usage and injuries through telephone interviews comprising 20 questions and discussion. Of the 3500 called, 383 parents generously contributed. This identified issues for a focused set of questions for the stage 3 survey, which involved sending emails to 14 784 participants (excluding those from stages 1 and 2), seeking their response to a comparatively brief questionnaire. A small incentive gave entry to a draw to win one of five trampoline ‘CarePacks’ valued at $59 each. There were 3817 respondents, a response rate of 26%, high for an email survey. Each stage required its own ethics approval.^15^

### Questionnaire

The emailed questions were:

1. When was your trampoline purchased? (0–1 year, 1–2 years, 2–3 years, 3+ years ago)
2. Has anyone been injured while using your trampoline? (Yes/No) (If ‘No’, the survey ended)
3. What was the gender of the injured person? (M/F)
4. What was the age of the person who was injured? (Age in years)
5. What was the injury? (Choices based on National Electronic Injury Surveillance System (NEISS) categories:^11^ strain or sprain, abrasion or contusion, laceration, fracture, head injury, internal injury, dental, dislocation, concussion, other (Free form))
6. What was the cause of injury? (Free form)
7. What treatment did the injured person receive? (Free form)
8. Where was treatment given? (at home, pharmacy/chemist, general practitioner/family doctor, ambulance/paramedic, emergency centre/hospital, specialist)

### Analysis of the responses

The analysis looked at the actual injuries and their causes (questions 5 and 6) and identified the proportion of injuries caused by features of the trampoline structure or design, compared with the proportion of injuries caused by the users to themselves, or to each other. Comparisons were made with similar analyses of injuries on conventional trampolines.

### Comparing the soft edge with Australian traditional trampolines

The injury cases from the stage 3 survey were grouped according to the three cause-categories used by Wallis *et al*. for their Australian study namely:^13^

**Table:** 

• Self and others	I hurt myself or was hurt by another person
• Fell off	I was hurt by falling off or hitting the ground
• Equipment/frame and springs	I was hurt by some feature of the equipment

**Fig. 2:**
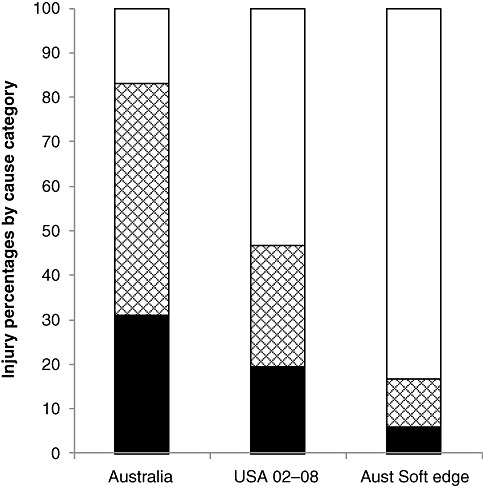
The nontraditional, soft-edge trampoline fell-off and equipment, frame & springs injuries are significantly lower than those of the US and Australian traditional trampolines. This also shows the difference between Australian and US injury proportions. (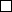) self and others, (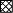) fell off, (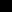) equipment/frame and springs.

Comparisons between the survey results and those from previous Australian data are given in Figure 2.

### Comparing the soft edge with both Australian and US traditional trampolines

Available US data on traditional trampolines from Alexander *et al*. was not in a form that could be directly compared with the three cause-categories previously, but it did distinguish five related categories, which were reorganised into the three categories noted in the previous section:^12^

- Hurt myself: Included in ‘self and others’ category
- Multiple jumpers: Included in ‘self and others’ category
- Fell off: Remains in the ‘fell-off’ category
- Equipment/frame and springs: Remains in same category
- Getting on or off: Small; removed and others scaled to suit

The US data are also shown in Figure 2.

#### Statistical treatment for comparisons with Australian and US data

A chi-square test for equality of three independent multinomial distributions was carried out to compare the distributions of injuries across this subset of three cause-categories between the US, Australian and soft-edge data sets. Similar, chi-square tests compared the soft-edged trampoline injury cause distributions with the Australian and US benchmarks.

### Analysis of causes of fell-off injuries in the soft-edge data

Although the soft-edge trampoline has an enclosure net, there were still a number of fell-off injuries. Individual cases were examined to determine the reasons for this.

### More serious injuries in the US data from traditional trampolines

To find whether the injuries caused by trampoline design were more serious than injuries caused by user behaviour, the analysis of the data from the US study was reopened by the authors, and the admissions proportion for injuries in the equipment/frame and springs and fell-off categories was compared with the emergency department presentation proportion of those categories.^12^ Comparison of the two showed whether admissions were disproportionately represented by the equipment/frame and springs and fell-off categories, and therefore represented more serious injuries. (Admissions chosen were NEISS ‘disposition codes’ 2, 4 and 5 in NEISS product code 1233 (trampolines).^11^)

### Overall injury reduction inferred from this study

**Fig. 3:**
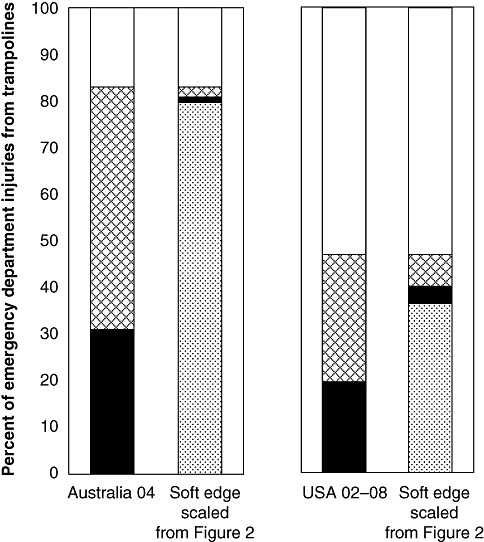
Indicated injury reduction percentages by the soft-edge trampoline compared with traditional trampolines, assuming injuries from ‘self and others’ will be equivalent on all trampoline types. (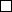) self and others, (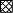) fell off, (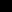) equipment/frame and springs, (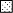) % injury reduction.

While this study has not directly measured how much the soft-edge trampoline can reduce injuries, a fair estimate can be made. On the assumption that trampoline users will hurt themselves and each other about the same rate on all trampolines regardless of type, then the ‘self and others’ categories in Figure 2 can be used as a reference to scale the soft-edge results, making the ‘self and others’ categories equivalent in each case and reducing the other categories in proportion. This leaves a deficit proportion indicative of the improvement made by the soft-edge design. This is shown pictorially in Figure 3.

### Age

**Fig. 4:**
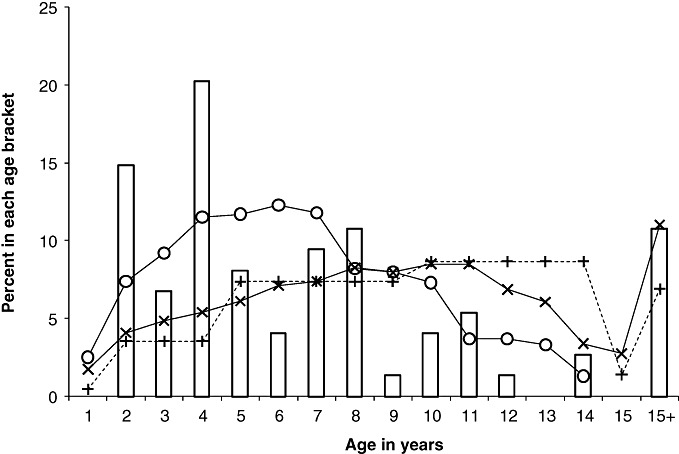
Age and distribution of reported injuries. Soft-edge data compare reasonably well with earlier Australian data which have a notably earlier age peak than US and Canadian data.^16^ (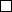) Soft-edge, 2010, (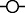) Australian data, 2004, QISU, Queensland Injury Surveillance Unit; (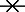) US data, 02–07, NEISS, (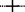) Canadian, 2006, CHIRPP, Canadian Hospitals Injury Reporting and Prevention Program.

All reported injuries were plotted against age in Figure 4 to show differences between data sets and countries.^11^,^16^,^17^

## Results

Of the 3817 respondents, 3417 reported no injury and 84 presented at an emergency centre/hospital or specialist; the 84 were used in comparisons with previous traditional trampoline data. Of those remaining, 34 were seen by a doctor, one by a paramedic and 281 were treated at home, and these three remaining sets were not used in comparisons.

### Comparing the soft edge with Australian traditional trampolines

Figure 2 shows the proportion of emergency department (or equivalent) presentations for each injury cause. A reduction in fell-off and equipment proportions is shown for the soft edge.

### Comparing the soft edge with both Australian and US traditional trampolines

Figure 2 shows that the soft-edged trampoline has lower proportions of fell-off and equipment/frame and springs injuries than traditional trampolines in both Australia and the USA. It also shows that the Australian data have a higher proportion of these two categories of injuries than the USA. (The US data is negligibly influenced by the soft-edged design which comprised about 0.4% of the US trampoline population at the time of the study.)

#### Statistical treatment of comparisons with Australian and US data

The chi-square test for equality of three independent multinomial distributions had a chi-squared test statistic of 72.3 on four degrees of freedom and *P*-value less than 0.001. This analysis confirms the visual impression in Figure 2 that the three injury cause distributions are different,

The chi-square tests comparing the soft-edged trampoline injury cause distributions with the Australian and US benchmarks also lead to *P*-values <0.001. This confirms the soft-edged trampoline has smaller proportions of injuries in the equipment/frame and springs and fell-off categories than the other data sets.

### Analysis of causes of fell-off injuries in the soft-edge data

Of the 40 cases of injuries from falling off, 36 reported the cause was the net door had not been zipped up.

### More serious injuries in the US data on traditional trampolines

The equipment/frame and springs and fell-off categories accounted for 55% (standard error (SE) 4.8) of all admissions (2002–2008). Because these two categories together only account for 46% (SE 1.1) of emergency department presentations, they are disproportionately represented in admissions, indicating these two cause-categories involve more serious injuries than the others.

### Overall injury reduction inferred from this study

Figure 3 shows that with the self and others categories used as a reference scale, the soft-edge trampoline is indicating a 36% reduction in injuries over traditional trampolines in the USA (in 2002–2008), and a 79% reduction over traditional trampolines in Australia (in 2004).

### Age

Soft-edge age data are compared in Figure 4 with traditional trampoline data from Australia, USA and Canada. The median age for injury is 5 for the soft-edge in this study, compared with 6 for Australian data on traditional trampolines. By contrast, the median age is 9 for children injured on traditional trampolines in both Canada and the USA.

## Discussion

The results in Figure 2 show that the soft-edged trampoline has a much lower proportion of equipment and fell-off injuries than traditionally designed trampolines in previous Australian and US studies.

The remaining injury causes, where children hurt themselves or each other, have been shown by US admissions data to result in less severe injuries than those caused by the equipment or by falling off. These remaining injuries are a consequence of user behaviour rather than hazardous equipment.

Strictly speaking an alternative interpretation of Figure 2 is that instead of a reduction in equipment injuries, there has been a large increase in self and others injuries. This type of study (changes in relative proportions) is not able to rule out this alternative perspective. The results of the questionnaire, however, do not support this alternative view. They show good parental awareness of the risks of multiple users and in no case do they suggest alarm that children hurt themselves or each other more on this than on any other trampoline type. It seems reasonable to assume that children's play will be broadly similar from trampoline to trampoline, and the large change in the proportions in Figure 2 is from improvements in the equipment as concluded previously.

Allowing this assumption, the inference from the results in Figure 3 is that an overall reduction in injuries on the soft-edge trampoline compared with the traditional version is something close to 80% for Australia and 35% for the USA.

The Australian, North American age comparison in Figure 4 shows a younger median age of about 5 or 6 for injuries in Australia. This is notable for two reasons: first, the trampoline standards for both countries specify that 6 years is the minimum age for users of these trampolines;^18^,^19^ second, Figure 2 shows much larger proportions of falling off and equipment injuries on traditional trampolines in Australia than in the USA. This difference may be to do with younger children on traditional trampolines in Australia, justifying the standard age restriction. Whether or not this difference between the USA and Australia is age related, the soft-edge data in Figures 2 and 3 shows an improvement over traditional trampolines in both countries.

A difficulty with this study was finding undeniably comparable data for the traditional trampoline. The relevant paper used was from 2004, 5 years before the present study.^13^ It has had to be assumed that little changed in that time. To help justify this assumption, the US study has been used for comparison as it specifically demonstrated that the causes of injuries on traditional trampolines remained constant from 2002 to 2007.^12^

An opportunity for this new design is the fact that almost all the fell-off injuries (36 out of 40) arose because users failed to zip up the door. While this is arguably a failure in user behaviour, it shows there is room for further improvement with an automatically closing door.

## Conclusions

This study helps validate the endeavour to design a safer trampoline, a long overdue constructive response to the many reports of increasing injury rates from domestic trampolines. It has shown that the soft-edge design has a significantly lower proportion of the more severe injuries comprising almost half of traditional trampoline injuries. The inferred improvements are an overall injury reduction of at least 30%. While not necessarily the only design with this profile, it is one where the evidence is available and it provides a benchmark for standards writers and designers. This information is important for agencies and decision-makers concerned with child safety and fitness.
